# Epidemiological and Clinical Insights into Enterovirus Circulation in Europe, 2018–2023: A Multicenter Retrospective Surveillance Study

**DOI:** 10.1093/infdis/jiaf179

**Published:** 2025-04-04

**Authors:** Sten de Schrijver, Emiel Vanhulle, Anne Ingenbleek, Leonidas Alexakis, Caroline Klint Johannesen, Eeva K Broberg, Heli Harvala, Thea K Fischer, Kimberley S M Benschop, ENPEN Study Collaborators, Jan Albert, Nancy Allen, Laurent Andreoletti, Sowsan Atabani, Christelle Auvray, Aldert Bart, Carla Berengua, Natasa Berginc, Maxime Bisseux, Hanneke Boon, Ana Bordes-Benitez, Andrew Bosworth, Sindy Böttcher, Maria Cabrerizo, Annapaola Callegaro, Cristina Calvo, Benjamin Canning, Marianne Coste-Burel, Karen Couderé, Jean-Marie Delarbre, Sabine Diedrich, Susanne Dudman, Robert Dyrdak, Julien Exinger, Eric Farfour, Maria Dolores Fernandez-Garcia, Jacky Flipse, Vincent Foulongne, Kristina Træholt Franck, Floriane Gallais, Magali Garcia, Irina Georgieva, Géraldine Gonfrier, Clémence Guillaume, Almudena Gutierrez-Arroyo, Cécile Henquell, Marijke Hooghiemstra, Elisabeth Huijskens, Anne J Jääskeläinen, Marion Jeannoël, Marine Jourdain, Elisabeth Toverud Landaas, Kathrin Keeren, Eduardo Lagarejos, Gisèle Lagathu, Sylvie Larrat, Cristian Launes, Mouna Lazrek, Caroline Lefeuvre, Véronique Lemée, Quentin Lepiller, Marianne Leruez, David Leyssene, Anne Sophie L'Honneur, Bruno Lina, Marie Louchet Ducoroy, Maja M Lunar, Jean-Michel Mansuy, Stéphanie Marque Julliet, Gina Mcallister, C Patrick McClure, Antonio Medina-Claros, Gregoria Megias, Sofie Elisabeth Midgley, Audrey Mirand, Richard Molenkamp, Milagrosa Montes, Antonio Moreno-Docón, Carmen Munoz-Almagro, Jean-Benjamin Murat, Jean-Luc Murk, Ana Navascues-Ortega, Maria Carmen Nieto-Toboso, Laetitia Ninove, Marije Oosting, Eider Oñate, Jordi Pacaud, Coralie Pallier, Elena Pariani, Laura Pellegrinelli, Mercedes Pérez-Ruiz, Sylvie Pillet, Léa Pilorgé, Luis Piñeiro, Mario Poljak, Birgit Prochazka, Emeline Riverain, Sylvie Rogez, Maud Salmona, Kenda Saloum, Erik Schaftenaar, Cécile Schanen, Manuel Schibler, Isabelle Schuffenecker, Karl Stefic, Petri Susi, Caroline M A Swanink, Charlotte Tellini, Anne Lise Toyer, Sara Colonia Uceda Renteria, Juan Valencia-Ramos, Freek B van Loenen, Inge van Loo, Véronique Venard, Jaco J Verweij, Karin J von Eije, Tytti Vuorinen, Elke Wollants, Laura Zanetti

**Affiliations:** Centre for Infectious Disease Control, Dutch National Public Health Institute, Bilthoven, The Netherlands; Centre for Infectious Disease Control, Dutch National Public Health Institute, Bilthoven, The Netherlands; European Program for Public Health Microbiology Training, European Centre for Disease, Prevention and Control, Stockholm, Sweden; European Centre for Disease Prevention and Control, Stockholm, Sweden; European Centre for Disease Prevention and Control, Stockholm, Sweden; Department of Clinical Research, Nordsjællands Hospital, Hillerød, Denmark; European Centre for Disease Prevention and Control, Stockholm, Sweden; National Health Service Blood and Transplant, Microbiology Services, Colindale, United Kingdom; Department of Clinical Research, Nordsjællands Hospital, Hillerød, Denmark; Department of Public Health, University of Copenhagen, Copenhagen, Denmark; Centre for Infectious Disease Control, Dutch National Public Health Institute, Bilthoven, The Netherlands

**Keywords:** enterovirus, laboratory detection, surveillance, Europe, epidemiology

## Abstract

**Background:**

Enteroviruses (EV) cause yearly outbreaks with severe infections, particularly in young children. This study investigates EV circulation, age, and clinical presentations in Europe from 2018 to 2023.

**Methods:**

Aggregated data were requested from the European Centre for Disease Prevention and Control National Focal Points for Surveillance and European Non-Polio Enterovirus Network. Data included detection month, specimen type, age group, and clinical presentation for the 10 most commonly reported EV types per year.

**Results:**

Twenty-eight institutions (16 countries) reported 563 654 EV tests during the study period with 33 265 (5.9%) EV positive. Forty-two types were identified (n = 11 605 cases) with echovirus 30 (E30), coxsackievirus A6 (CVA6), EV-D68, E9, E11, CVB5, E18, CVB4, EV-A71, and E6 most frequently reported. E30 declined after 2018/2019, while CVA6, CVB5, E9, E11, and EV-D68 were prevalent both before and after the coronavirus disease 2019 (COVID-19) pandemic, and CVB4 and E18 were prevalent after the pandemic. A shift in seasons (summer to fall) and specimen positivity (feces to respiratory) was observed. Neurological signs predominated among EV-A71, CVB4, CVB5, E6, E9, E11, E18, and E30 (30%–72%). CVB4, CVB5, E9, E11, and E18 were frequently reported among neonates (18%–32%). CVA6 was frequently associated with hand, foot and mouth disease, and EV-D68 with respiratory infections. Paralysis was reported among 22 infections, associated with 10 nonpolio types.

**Conclusions:**

This study emphasizes the widespread circulation and severity of EV infections in Europe, as well as the (re)emergence of specific types postpandemic. Our findings highlight the need for continuous EV surveillance to monitor variation in circulation, age, and clinical presentations, including paralysis among nonpolio EV infections.

## Teaser

This study provides insight into the changing epidemiological landscape of (re)emerging enteroviruses in Europe in 2018–2023 with severe clinical presentations like neurological infections/paralysis. Echovirus (E)30, coxsackievirus (CV)A6, EV-D68, E9, E11, CVB5, E18, CVB4, EV-A71, and E6 were most frequently reported.

Enteroviruses (EVs) are a diverse group of nonenveloped, single-stranded RNA viruses belonging to the genus *Enterovirus* within the Picornaviridae family [1]. To date, over 110 EV types that infect humans have been classified into species A–D [2].

EVs are primarily transmitted via the fecal-oral and respiratory routes [3–5]. They pose a significant public health threat with a broad range of severe clinical manifestations, including hand, foot, and mouth disease (HFMD), respiratory illnesses, meningitis, paralysis, and encephalitis [3, 4, 6], particularly in newborns and young children [4, 5, 7]. Following the coronavirus disease 2019 (COVID-19) pandemic, outbreaks of echovirus 11 (E11), E18, coxsackievirus B types (CVBs), and EV-D68 have been reported with severe clinical outcomes such as hepatitis, meningitis, myocarditis, and paralysis, respectively, predominantly affecting neonates and young children [8–13].

Although surveillance has proven to be essential in contributing to poliovirus elimination and vaccination programs, nonpolio EV surveillance is passive and remains substantially inconsistent across Europe. With the lack of implemented surveillance systems, the European Non-Polio Enterovirus Network (ENPEN) was established to standardize data collection of EV infections, to monitor EV circulation, and to estimate the disease burden of these viruses across Europe [3, 14]. In this study, we investigate EV epidemiological and clinical patterns, such as seasonality, specimen type, age distribution, and clinical presentations, and the (re)emergence of specific EV types in Europe from 2018 through 2023.

## METHODS

### Study Participation

An invitation to participate in this study was sent (25 August 2023) to all institutions within ENPEN and the European Centre for Disease Prevention and Control (ECDC) National Coordinators of the Competent Bodies, responsible for overall coordination of public health interactions in the Member States within the European Union and European Economic Area (EU/EEA). Additionally, requests were sent to the ECDC National Focal Points for Surveillance; Microbiology; Preparedness and Response; and Threat Detection. Contacted representatives were asked to forward the invitation to other local or regional public health officials potentially interested in participating.

### Data Collection

Data were recorded in an aggregated format and collected using a secured ECDC-ENPEN study collaboration portal hosted at ECDC with a data collection form (Supplementary Material). All reporting institutions were assigned a code, comprising of the 2-letter country code (ISO 3166-1 alpha-2 codes) and a sequential number (Supplementary Tables 1 and 2). Aggregated testing and typing data were collected from EV tested cases, presenting in hospital settings of participating institutes. These data included the number of clinical samples tested for EV, the number of EV-positive samples, the number of samples subjected to EV typing, and the number successfully typed, covering the study period from January 2018 through August 2023 (Supplementary Table 2). In addition, each institution was requested to supply data stratified by EV type and year on the monthly distribution of infections, patient age groups, specimen type tested, and clinical presentations (eg, fever, gastrointestinal symptoms, or neurological infections) (Supplementary Table 2) of the 10 most detected EV types. Institutions were asked to provide information on the method used for EV testing and typing. Sequence data and information on rhinovirus and parechovirus testing were not collected as part of this study.

### Data Analysis and Statistics

A retrospective descriptive analysis of the reported data was performed by EV type for each country, as well as aggregated totals by month, year, specimen type, age group, and clinical presentation. To identify EV-type–specific patterns, these variables were normalized per type. Seasons were defined in 3-month intervals (eg, June–August for summer and September–November for fall). Statistical significance was assessed using the χ^2^ test for independence, with a *P* value <.05 considered as significant. Data analysis and visualization were performed in RStudio (version 2023.12.1.402). Analysis scripts are publicly available: https://github.com/ENPEN-RIVM/Retrospective-surveillance-study.

## RESULTS

### Enterovirus Testing and Typing

**Figure 1.**
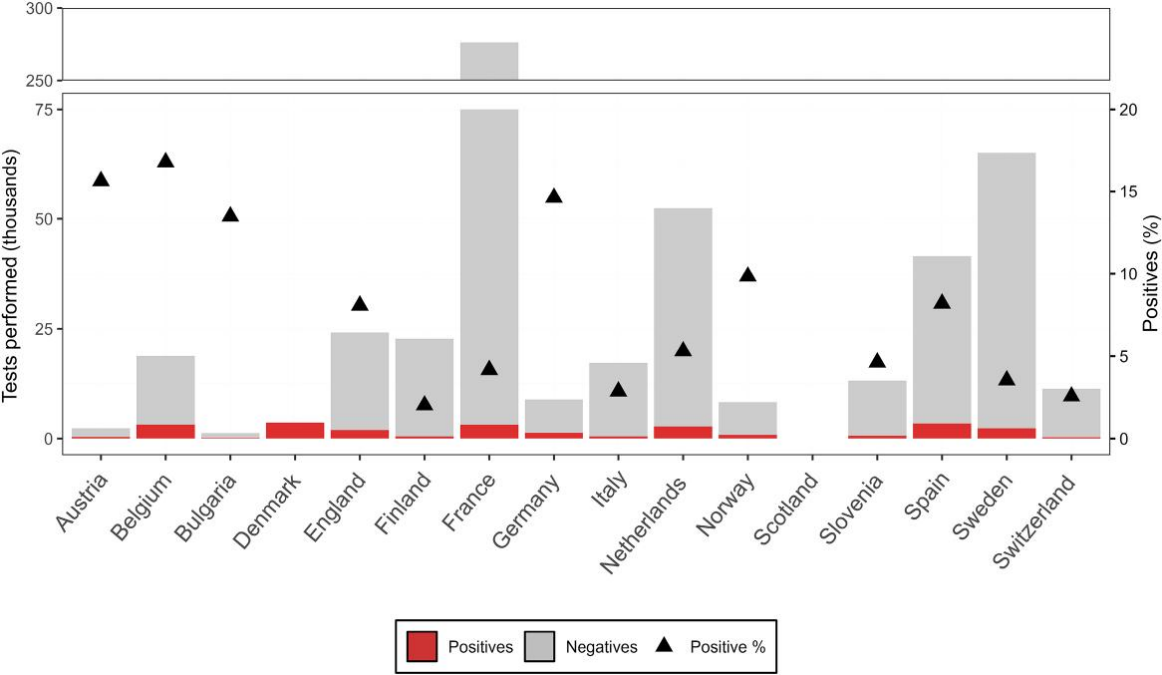
Enterovirus (EV)-positive samples per participating country. Total number of EV-positive and -negative tests reported, along with the positivity rate (black triangle), between 2018 and 2023 (n = 563 654). No testing data were reported by Scotland, and no data on the total number of tests were reported from Denmark.

A total of 28 institutions from 16 European countries responded to the invitation (Supplementary Figure 1 and Supplementary Tables 1 and 2). The common method for EV typing was VP1 sequencing (27/28 institutions) [15]. One institution relied on virus neutralization for typing, although a selection of samples was subjected to VP1 sequencing (Supplementary Table 2). Information on type-specific testing was not consistently reported. Over the study period, in total 563 654 clinical samples were tested for EV, of which 33 265 (5.9%) were EV positive. EV-positive rates varied among countries (2.0% in Finland to 17% in Belgium; Figure 1) and among institutes (Supplementary Table 1). Of the EV-positive samples, 21 268 (63.9%) were subjected to typing, with 17 081 (80.3%) samples successfully typed and epidemiological and clinical data reported for 11 605 of these typed samples (Supplementary Figure 1 and Supplementary Table 1). However, the proportion of samples selected for typing and the typing success rate varied across institutions (Supplementary Table 1).

### Diversity of Enterovirus Types

**Figure 2.**
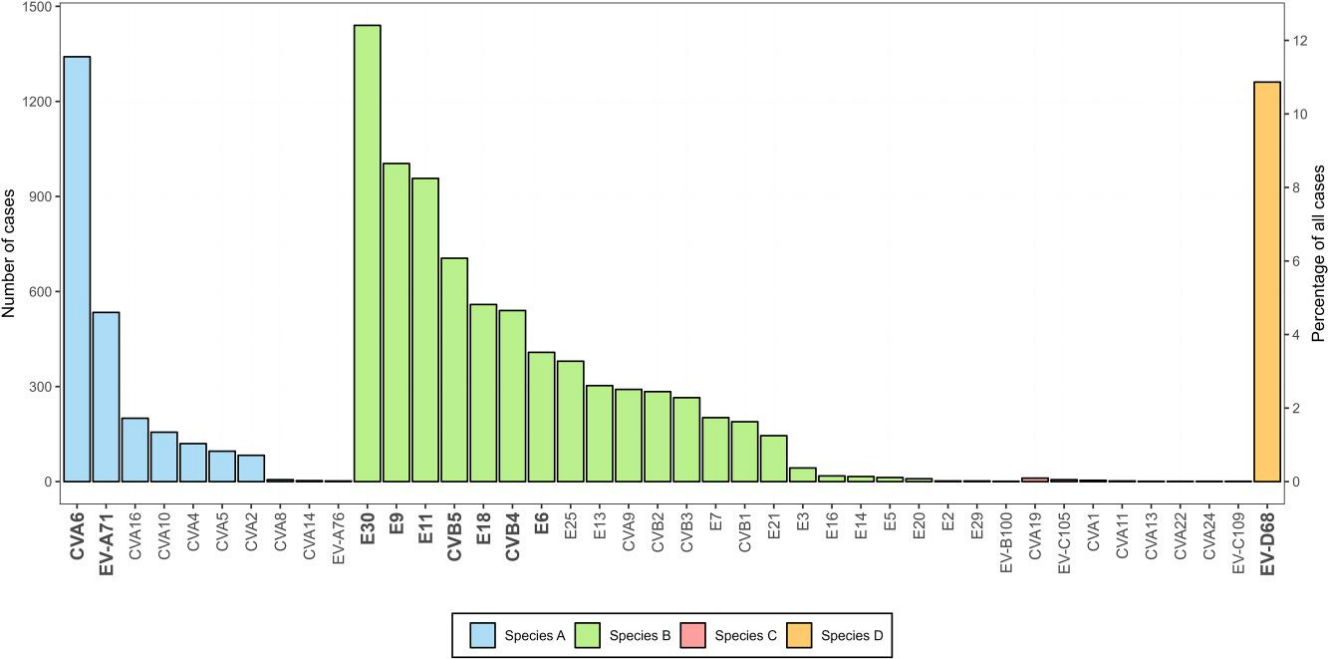
Enterovirus (EV) type distribution. The overall number of cases associated with each detected EV type between 2018 and 2023 (n = 11 605). The 10 most commonly reported EV types among all participants are indicated in bold. Abbreviations: CV, coxsackievirus; E, echovirus; EV, enterovirus.

Between 2018 and 2023, 42 enterovirus types were reported among 11 605 infections (Figure 2 and Supplementary Figure 1). Most types belonged to EV-B (23 types, n = 7776 infections), followed by EV-A (10 types, n = 2541 infections), EV-C (8 types, n = 27 infections), and EV-D (EV-D68, n = 1261 infections). The most frequently reported types were E30 (n = 1440), CVA6 (n = 1341), EV-D68 (n = 1261), E9 (n = 1004), E11 (n = 957), CVB5 (n = 705), E18 (n = 559), CVB4 (n = 540), EV-A71 (n = 534), and E6 (n = 408). Together, these 10 types accounted for 8749 infections (75.4%) (Figure 2 and Table 1). Although no particular EV-C type was associated with a high case count, the diversity of types detected was notable, with CV-A19 (n = 11), EV-C105 (n = 6), CV-A1 (n = 4), CV-A11 (n = 2), and single detections of CV-A13, CV-A22, CV-A24, and EV-C109. Most EV-C detections, including an EV-B100, were detected in 2021–2023. Interestingly, EV-C infections, for which age was known (n = 18/21), were detected in older age groups (>1 year) (data not shown).

**Table 1.** **Detection of the 10 Most Commonly Reported Enterovirus** (**EV) Types, per Year, 2018–2023**

EV Type	Total Reported		2018		2019		2020		2021		2022		2023 to August	
	n	(%)	n	(%)	n	(%)	n	(%)	n	(%)	n	(%)	n	(%)
E30	1440	(12.4)	1076	(31.8)	357	(13.2)	5	(1.2)	0	(0.0)	0	(0.0)	2	(0.2)
CVA6	1341	(11.6)	340	(10.0)	254	(9.4)	86	(20.7)	350	(27.1)	203	(7.5)	108	(9.8)
EV-D68	1261	(10.9)	227	(6.7)	52	(1.9)	43	(10.3)	336	(26.0)	581	(21.5)	22	(2.0)
E9	1004	(8.7)	408	(12.0)	119	(4.4)	33	(7.9)	38	(2.9)	240	(8.9)	166	(15.0)
E11	957	(8.2)	262	(7.7)	175	(6.5)	15	(3.6)	30	(2.3)	342	(12.6)	133	(12.0)
CVB5	705	(6.1)	74	(2.2)	255	(9.4)	6	(1.4)	107	(8.3)	212	(7.8)	51	(4.6)
E18	559	(4.8)	120	(3.5)	17	(0.6)	37	(8.9)	63	(4.9)	97	(3.6)	225	(20.4)
CVB4	551	(4.7)	43	(1.3)	88	(3.3)	2	(0.5)	30	(2.3)	354	(13.1)	23	(2.1)
EV-A71	534	(4.6)	131	(3.9)	271	(10.0)	9	(2.2)	5	(0.4)	49	(1.8)	69	(6.3)
E6	408	(3.5)	180	(5.3)	104	(3.8)	16	(3.8)	4	(0.3)	37	(1.4)	67	(6.1)
Other types	2856	(24.6)	525	(15.5)	1010	(37.4)	164	(39.4)	330	(25.5)	589	(21.8)	238	(21.6)
Total top 10	8749	(75.4)	2861	(84.5)	1692	(62.6)	252	(60.6)	963	(74.5)	2115	(78.2)	866	(78.4)
Total all types	11605	…	3386	…	2702	…	416	…	1293	…	2704	…	1104	…

### Temporal and Seasonal Dynamics

**Figure 3.**
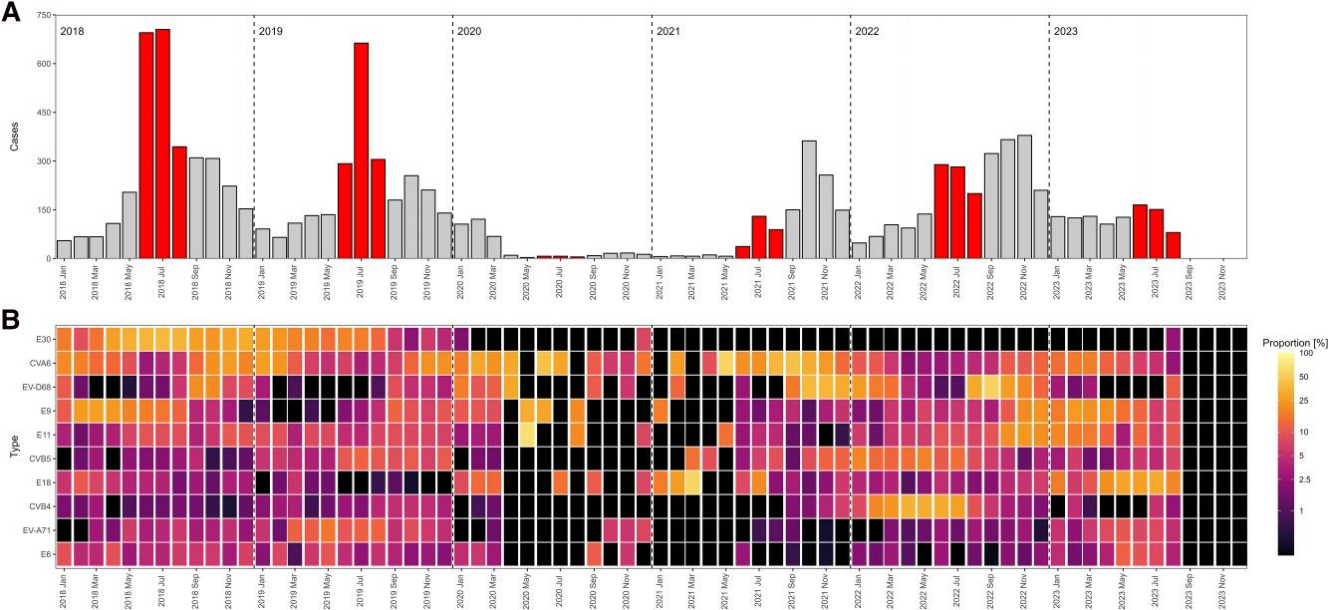
*A*, Temporal distribution of EV-positive cases, 2018–2023 (n = 10 925). The summer months (June-August) are highlighted in red. *B*, Relative monthly distribution of 10 most commonly reported EV types. The color intensity in the heat maps is based on the natural logarithm of the fraction of the number of cases of each type over the total number of cases within each month adding up to 100%. Black regions represent either zero infections or no data. Abbreviations: CV, coxsackievirus; E, echovirus; EV, enterovirus.

The detection month was reported for 10 925 infections (94.1%) across 13 countries (Table 1, Supplementary Figure 1, and Supplementary Table 2). A shift from summer to fall predominance is observed after 2021 (Figure 3).

During 2018 and 2019, the majority of infections occurred in the summer with 1744 infections (51.5% of 3386 infections) in 2018 and 1260 infections (46.6% of 2702 infections) in 2019. E30 dominated during these years, accounting for 31.8% (n = 1076) of all typed infections in 2018 and 13.2% (n = 357) in 2019 (Table 1). However, E30 detection steadily declined after spring 2019, with only 2 infections in August 2023 (Figure 3).

In 2020 and early 2021 a decline in reported infections was observed; 416 infections in 2020 and 295 infections until August 2021 (Figure 3 and Table 1). A resurgence of EV infections was observed in fall 2021 (n = 998, 77.2% of all 2021 infections), surpassing the summer peak (256 infections, 19.8%). This seasonal shift persisted into 2022, with fall accounting for 1068 infections (39.5%) compared to 771 infections (28.5%) in summer. Despite limited data for fall 2023, a decline in infections was observed in summer (n = 396), as seen in previous years.

The 2021 fall peak was predominantly attributed to CVA6 (n = 244 of 350 [69.7%] infections) and EV-D68 (n = 255 of 336 [75.9%] infections) (Figure 3 and Table 1), while the fall peak of 2022 was driven by increased detections of EV-D68 (n = 344 of 581 [59.1%] infections) (Figure 3 and Table 1). Both CVA6 and EV-D68 had notable increased detection in the fall and winter months before and after the COVID-19 pandemic (Figure 3 and Table 1). Of note, EV-D68 detection rates in 2021–2022 were higher than in 2018–2019 (Table 1).

Other noteworthy trends include the increased detection of E9 with 408 infections in 2018 (12.0%), 119 infections in 2019 (4.4%), and 240 infections in 2022 (8.9%). The same trend was observed for E11 in 2018 (n = 262, 7.7%), 2019 (n = 175, 6.5%), and 2022 (n = 342, 12.6%) (Figure 3 and Table 1). Similarly, CVB5 was increasingly detected in the years before and after the COVID-19 pandemic with 225 infections in 2018 (9.4%) and 212 infections in 2022 (7.8%).

CVB4 and E18 showed increased detection only after the pandemic with 354 infections (13.1%) in 2022. CVB4 was predominantly reported in the spring/summer of 2022 (n = 285, 80.1%). E18 emerged as the dominant type during summer 2023, accounting for 107 of the 225 (47.6%) E18 infections that year (Figure 3 and Table 1). EV-A71 was only reported with increased numbers in 2019 (n = 271, 10.0%).

### Trends in EV-Positive Specimen Types

**Figure 4.**
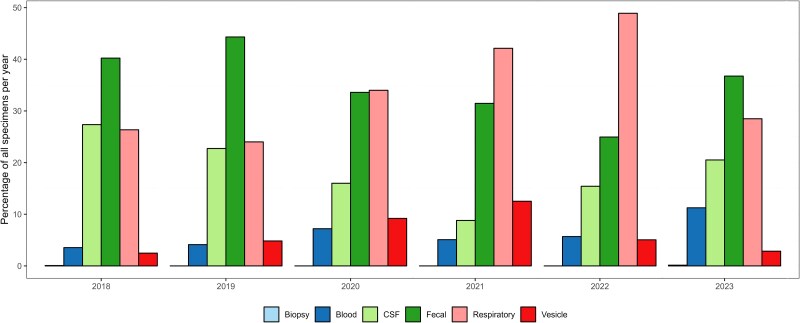
Distribution of specimen types among enterovirus-positive samples per year, 2018–2023 (n = 10 925).

Among the successfully typed EV-positive samples, specimen type data were reported for 5878 samples (50.7%) by 12 countries (Table 2, Supplementary Figure 1, and Supplementary Table 1). Feces was the most frequently reported specimen type (n = 2111, 35.9%), followed by respiratory samples (n = 1965, 33.4%). A shift in predominant specimen type was observed over time, with fecal samples being most reported in 2018 and 2019, whereas respiratory samples became more frequently detected in 2020 (n = 84, 34.0%), 2021 (n = 249, 42.1%), and 2022 (n = 698, 48.9%) (Figure 4). While data for 2023 covers only up to August, thereby omitting the fall/winter season, fecal samples once again predominated in 2023 (n = 258, 37% fecal vs n = 200, 28.5% respiratory). A statistically significant correlation was identified between the year and distribution of specimen types tested (*P* < .0001). Specimen type distribution also varied among institutions, with feces reported as the dominant positive specimen in 9 institutes (5 countries), and respiratory samples being most frequently detected in 7 institutes (6 countries) (Supplementary Table 2).

**Table 2.** Detection of the 10 Most Commonly Reported EV Types by Specimen, Type Patient Age, and Clinical Presentation

	10 Most Commonly Reported EV Types																							
	E30		CVA6		EV-D68		E9		E11		CVB5		E18		CVB4		EV-A71		E6		Other Types		Total Types	
	n	(%)	n	(%)	n	(%)	n	(%)	n	(%)	n	(%)	n	(%)	n	(%)	n	(%)	n	(%)	n	(%)	n	(%)
Specimen type																								
Biopsy	NA	…	NA	…	1	(0.1)	NA	…	NA	…	NA	…	NA	…	NA	…	NA	…	1	(0.3)	0	…	2	(0.03)
Blood	7	(1.2)	33	(5.2)	1	(0.1)	33	(8.4)	36	(7.9)	10	(2.7)	44	(18.1)	26	(8.9)	12	(3.4)	6	(2.0)	111	(7.3)	319	(5.4)
CSF	280	(49.9)	6	(0.9)	1	(0.1)	141	(35.8)	98	(21.4)	81	(21.7)	57	(23.5)	46	(15.8)	18	(5.0)	149	(50.3)	310	(20.4)	1187	(20.2)
Fecal	240	(42.8)	128	(20.2)	11	(1.5)	115	(29.2)	192	(42.0)	218	(58.4)	108	(44.4)	125	(42.8)	232	(64.8)	84	(28.4)	658	(43.2)	2111	(35.9)
Respiratory	34	(6.1)	216	(34.1)	731	(97.6)	102	(25.9)	130	(28.4)	64	(17.2)	34	(14.0)	95	(32.5)	90	(25.1)	56	(18.9)	413	(27.1)	1965	(33.4)
Vesicle	NA	…	250	(39.5)	4	(0.5)	3	(0.8)	1	(0.2)	NA	…	NA	…	NA	…	6	(1.7)	NA	…	30	(2.0)	294	(5.0)
Total	561	…	633	…	749	…	394	…	457	…	373	…	243	…	292	…	358	…	296	…	1522	…	5878	…
Age																								
≤ 28 d	150	(10.8)	20	(1.6)	28	(2.3)	170	(18.4)	266	(31.1)	156	(24.3)	119	(24.2)	152	(32.1)	83	(16.9)	34	(9.2)	606	(24.0)	1784	(16.8)
29 d–3.0 mo	159	(11.5)	49	(3.9)	91	(7.5)	170	(18.4)	225	(26.3)	126	(19.6)	134	(27.3)	90	(19.0)	98	(20.0)	25	(6.7)	11	(0.4)	1178	(11.1)
3.1–6 mo	10	(0.7)	48	(3.9)	52	(4.3)	23	(2.5)	41	(4.8)	12	(1.9)	10	(2.0)	17	(3.6)	21	(4.3)	14	(3.8)	698	(27.7)	946	(8.9)
7–12 mo	15	(1.1)	155	(12.4)	88	(7.3)	25	(2.7)	54	(6.3)	20	(3.1)	8	(1.6)	15	(3.2)	36	(7.3)	4	(1.1)	51	(2.0)	471	(4.4)
1–5 y	205	(14.8)	695	(55.8)	569	(47.2)	169	(18.3)	155	(18.1)	137	(21.3)	80	(16.3)	122	(25.8)	200	(40.7)	115	(31.0)	219	(8.7)	2666	(25.1)
6–15 y	311	(22.5)	45	(3.6)	154	(12.8)	165	(17.9)	42	(4.9)	59	(9.2)	48	(9.8)	33	(7.0)	38	(7.7)	70	(18.9)	530	(21.0)	1495	(14.1)
16–25 y	141	(10.2)	37	(3.0)	27	(2.2)	49	(5.3)	14	(1.6)	19	(3.0)	20	(4.1)	5	(1.1)	2	(0.4)	26	(7.0)	77	(3.1)	417	(3.9)
26–45 y	353	(25.5)	147	(11.8)	65	(5.4)	148	(16.0)	57	(6.7)	100	(15.6)	71	(14.5)	39	(8.2)	9	(1.8)	77	(20.8)	21	(0.8)	1087	(10.2)
46–65 y	32	(2.3)	38	(3.0)	68	(5.6)	4	(0.4)	1	(0.1)	6	(0.9)	1	(0.2)	NA	…	3	(0.6)	6	(1.6)	186	(7.4)	345	(3.3)
> 65 y	7	(0.5)	12	(1.0)	64	(5.3)	NA	…	1	(0.1)	7	(1.1)	NA	…	NA	…	1	(0.2)	NA	…	125	(5.0)	217	(2.0)
Total	1383	…	1246	…	1206	…	923	…	856	…	642	…	491	…	473	…	491	…	371	…	2524	…	10606	…
Clinical presentation																								
Fever	48	(16.8)	29	(7.8)	20	(5.6)	86	(38.1)	90	(33.5)	17	(9.0)	62	(48.1)	41	(29.5)	27	(10.4)	29	(18.2)	249	(30.6)	698	(21.9)
Gastrointestinal	5	(1.8)	4	(1.1)	2	(0.6)	6	(2.7)	10	(3.7)	4	(2.1)	2	(1.6)	2	(1.4)	3	(1.2)	5	(3.1)	43	(5.3)	86	(2.7)
Hepatitis	NA	…	NA	…	NA	…	NA	…	1	(0.4)	NA	…	NA	…	NA	…	NA	…	NA	…	3	(0.4)	4	(0.1)
HFMD	NA	…	148	(40.0)	NA	…	2	(0.9)	NA	…	NA	…	NA	…	NA	…	NA	…	1	(0.6)	15	(1.8)	166	(5.2)
Myocarditis	NA	…	NA	…	1	(0.3)	1	(0.4)	NA	…	1	(0.5)	1	(0.8)	3	(2.2)	1	(0.4)	NA	…	4	(0.5)	12	(0.4)
Neonatal sepsis	3	(1.1)	1	(0.3)	NA	…	NA	…	13	(4.8)	4	(2.1)	5	(3.9)	2	(1.4)	1	(0.4)	3	(1.9)	18	(2.2)	50	(1.6)
Neurological	204	(71.6)	56	(15.1)	3	(0.8)	98	(43.4)	114	(42.4)	137	(72.9)	39	(30.2)	63	(45.3)	189	(73.0)	90	(56.6)	301	(37.0)	1294	(40.5)
Other rash, non-HFMD	NA	…	51	(13.8)	NA	…	5	(2.2)	4	(1.5)	NA	…	2	(1.6)	1	(0.7)	3	(1.2)	2	(1.3)	12	(1.5)	80	(2.5)
Paralysis	NA	…	2	(0.5)	4	(1.1)	1	(0.4)	2	(0.7)	NA	…	5	(3.9)	1	(0.7)	3	(1.2)	NA	…	4	(0.5)	22	(0.7)
Respiratory	25	(8.8)	79	(21.4)	325	(91.5)	27	(11.9)	35	(13.0)	25	(13.3)	13	(10.1)	26	(18.7)	32	(12.4)	29	(18.2)	165	(20.3)	781	(24.5)
Total	285	…	370	…	355	…	226	…	269	…	188	…	129	…	139	…	259	…	159	…	814	…	3193	…

Abbreviations: CSF, cerebrospinal fluid; EV, enterovirus; HFMD, hand, foot and mouth disease; NA, not applicable.

**Figure 5.**
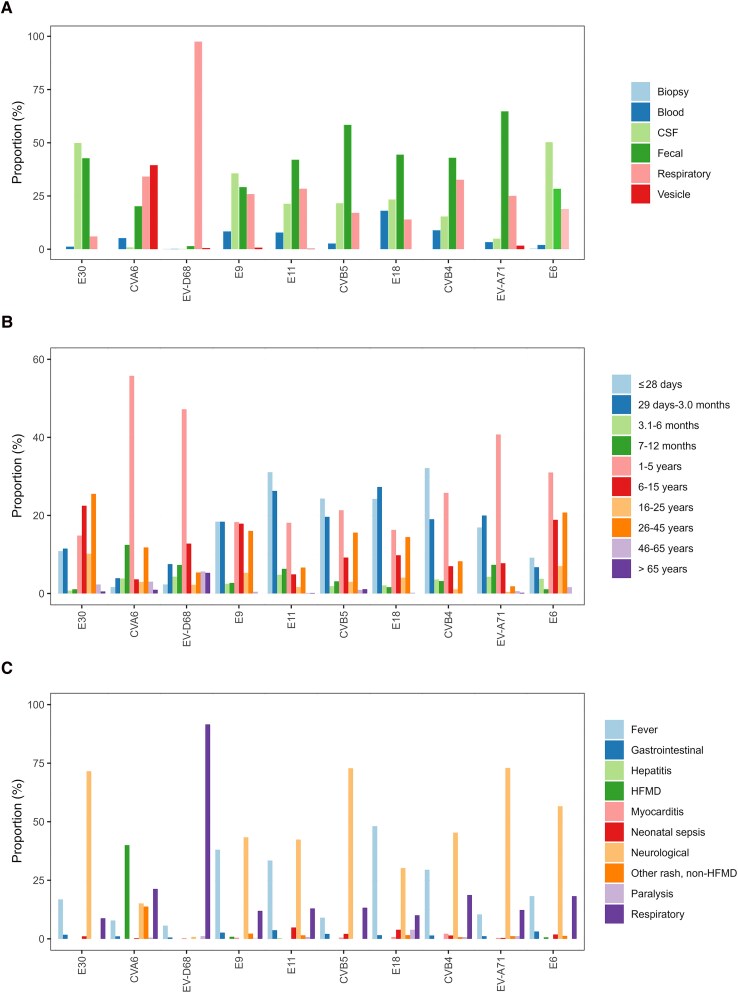
EV detection patterns of the 10 most commonly reported EV types by (*A*) specimen type (n = 4355), (*B*) age group (n = 8082), and (*C*) clinical presentation (n = 2379), normalized per type, between 2018 and 2023. Abbreviations: CSF, cerebrospinal fluid; CV, coxsackievirus; E, echovirus; EV, enterovirus; HFMD, hand, foot, and mouth disease.

Feces was the primary source for EV-A71, accounting for 64.8% of detections with this type, followed by CVB4 (42.8%), CVB5 (58.4%), E11 (42.0%), and E18 (44.4%) (Figure 5*A* and Table 2). This trend remained consistent throughout the study period (Supplementary Figure 2). CVA6 was primarily detected in vesicle swabs (n = 250, 39.5%), while E30, E6, and E9 were predominantly detected in cerebrospinal fluid (CSF) (n = 280 [49.9%], n = 149 [50.3%], and n = 141 [35.8%], respectively). EV-D68 was almost exclusively identified in respiratory samples (n = 731, 98%). The pattern remained consistent over the years where variations depended on which type dominated in a given year (Supplementary Figure 2).

### Age-Related Patterns

Patient age was reported for 10 606 infections (91.4%) across 13 countries (Table 2, Supplementary Figure 1, and Supplementary Table 1). Clear age-specific distributions were observed among the 10 most commonly reported EV types. E9, E11, E18, CVB4, and CVB5 were predominantly reported in neonates (aged ≤28 days), accounting for 863 infections (48.4%), and children aged 1–3 months (n = 745, 41.8%) In contrast, CVA6, EV-D68, EV-A71, and E6 were frequently reported in the 1–5 year age group (n = 1576, 59.2%; Figure 5*B* and Table 2). E6 infections were also frequently found among school-aged children 6–15 years old (n = 70, 18.9%) and adults aged 26–45 years (n = 77, 20.8%). E30 showed a distinct pattern, being most prevalent in individuals aged 6–15 years (n = 311, 22.5%) and adults aged 26–45 years (n = 353, 25.5%) (Figure 5*B* and Table 2). The age-related pattern remained consistent over the years where variations depended on which type dominated in a given year (Supplementary Figure 2).

### Diverse Clinical Manifestations

The clinical presentation was reported for 3193 infections (27.5%) from 8 countries (Supplementary Figure 1, Table 2, and Supplementary Table 1). Neurological infections were a frequently reported clinical presentation (n = 1294, 40.5%), followed by respiratory infections (n = 781, 24.5%) and fever (n = 698, 21.9%) (Table 2). Certain types showed strong association with neurological infections, including EV-A71 (n = 189, 73%), CV-B5 (n = 137, 72.9%), E30 (n = 204, 71.6%), E6 (n = 90, 56.6%), CVB4 (n = 63, 45.3%), E9 (n = 98, 43.4%), E11 (n = 114, 42.4%), and E18 (n = 39, 30.2%). Respiratory infections were predominantly associated with EV-D68 (n = 325, 91.5%), while CVA6 was frequently associated with HFMD (n = 148, 40%) (Figure 5*C*, Table 2, and Supplementary Figure 2). Of note, hepatitis was rarely reported, with only 4 infections documented in 2022 (E7, E11, E21, and CVB3). Myocarditis was reported in 12 infections, 3 of which were CVB4 infections (Table 2).

Although the clinical presentations remained consistent over the years, variations in reported infections reflected fluctuations in the predominant circulating types. Interestingly, paralysis was documented among 22 infections, associated with 10 different nonpolio EV types: E18 (n = 5), EV-D68 (n = 4), EV-A71 (n = 3), CVA6 (n = 2), CVA9 (n = 2), E11 (n = 2), CVB4 (n = 1), E3 (n = 1), E9 (n = 1), and E25 (n = 1) (Figure 5*C*, Table 2, and Supplementary Figure 2).

## DISCUSSION

Here we investigate EV epidemiological and clinical patterns, such as seasonality, specimen type, age distribution, and clinical presentations, and the (re)emergence of specific EV types in Europe from 2018 through 2023. We report more than half a million tests, 33 265 EV-positive samples with 21 268 (63.9%) subjected to typing, and 17 081 (80.3%) samples successfully typed. Epidemiological and clinical data were provided for each institute's 10 most commonly typed EV, accounting for 11 605 infections that were successfully typed, encompassing 1291 neurological infections (40.5% of 10 606 reported infections with clinical information) and 22 nonpolio enterovirus infections associated with paralysis.

The study provides novel insights into the evolving characteristics of EV types in Europe. Most importantly, the study stresses the critical need for vigilant monitoring of severe EV infections, particularly in vulnerable age groups.

In total, 42 different EV types were detected, with E30, CVA6, EV-D68, E9, E11, CVB5, E18, CVB4, EV-A71, and E6 the most frequently reported types, each with type-specific temporal, age related, and clinical infections. Interestingly, a higher number of rare EV-C types were reported compared to previous studies [7, 16]. However, it is important to note that institutes only provided data on their 10 most commonly detected types. As such, institutions with larger datasets are less likely to report rare EVs. This likely results in an underestimation of their true incidence. Harvala et al previously documented an abundance of EV-C types, although exclusively in sewage sampling [16]. In contrast, our findings indicate the presence of EV-C types in clinical samples, predominantly postpandemic and in older age groups, suggesting potential changes in their pathogenicity and/or population immunity. However, this observation is based on a small number of cases (n = 21) and hence should be interpreted with caution. Strikingly, a transition from summer to fall predominance among the 10 most commonly reported types was observed after 2020, coinciding with increased positivity of respiratory specimens. The 2021 fall peak was characterized by increased detections of CVA6 and EV-D68 and in the fall of 2022 by EV-D68. Both viruses are notable for their activity during these seasons [7, 8, 17–20]. The seasonal shift could potentially be caused by increased detection of specific types in a given year, although social and behavioral patterns, changes in population immunity, or heightened susceptibility to respiratory pathogens in the postpandemic context can also potentially drive this seasonal change. Increased testing of respiratory specimens during and after the pandemic due to severe acute respiratory syndrome coronavirus 2 (SARS-CoV-2) testing using multirespiratory pathogen detection assays that include EVs may have facilitated the detection of respiratory-associated EV types like EV-D68. Here we did not collect data on specimen types tested per year and more standardized data on testing practices are needed to study this shift.

Analysis of the 10 most commonly reported EV types over the study period demonstrates remarkable patterns in their epidemiology. Our study reports a higher number of EV-D68 infections compared to previous years [7]. EV-D68 was frequently reported among cases with respiratory infections and, not surprisingly, it was frequently found among respiratory specimens. Historically, EV-D68 infections have been associated with sporadic outbreaks occurring in a biennial pattern, with peaks during the fall of even-numbered years [17, 20]. However, our findings suggest a departure from this pattern, with yearly circulation, after 2021 (Figure 3) [8, 21]. This warrants vigilance, particularly regarding the occurrence of paralysis cases, often associated with EV-D68 infections. Interestingly, the seasonal shift seen in EV-D68 detections aligns with changes observed in other respiratory pathogens, such as respiratory syncytial virus, also displaying altered seasonality in recent years [22]. These findings indicate the importance of ongoing surveillance to monitor evolving trends in EV-D68 circulation and their implications for public health.

Like EV-D68, CVA6, was predominantly reported in the fall-winter months. This type was also predominantly reported among HFMD infections, with vesicle fluid as the dominant sample type of detection. Knowledge of its fall dominance and increased detections in the last years [7] enables preparedness in the fall for clinical care of HFMD cases.

The E30 epidemiology observed reflects the occurrence of a quiescent period following increased detection during the 2018–2019 outbreak [23, 24]. E30 is known to follow characteristic cyclical epidemiological patterns, with outbreaks occurring approximately every 3–5 years, often driven by the emergence of recombinant strains [23, 25, 26]. The 2018 outbreak was previously linked to the emergence of a recombinant strain and a new immune-divergent variant [23, 24]. Consistent with its profile as a neurological enterovirus [24, 27–29], our study shows that 72% of E30 infection were associated with neurological infections, and CSF was the dominant specimen type from which E30 was detected (50%). Interestingly, E30 infections were more often reported among older children (6–15 years old, 22%), and young adults (26–45 years old, 26%), contrasting with the typical younger age distribution of enterovirus infections [4, 7]. Given E30's periodic circulation and the long-observed interval of low/silent circulation, it is expected that this type may reemerge in the coming years, potentially associated with neurological infections. To monitor the emergence of new recombinant strains, advanced molecular techniques such as next generation sequencing, which enable complete genome analyses and recombination detection, could play a role in surveillance efforts.

In contrast to E30, EV types CVA6, CVB5, E9, E11, and EV-D68 were prevalent both before and after the COVID-19 pandemic, while CVB4 and E18 were more prevalent after the pandemic. Like E30, EV types CVB4, CVB5, E9, E11, and E18 were commonly associated with neurological infections, but they predominantly affected neonates (aged ≤28 days, 18%–32%) and infants aged 1–3 months (18%–27%). This could be related to an extended herd immunity gap after the pandemic placing neonates at increased risk for severe infections [30].

Overall, most samples isolated from patients presenting with neurological infections contained EV-B types (76%), similar to the study of Bubba et al [7]. The CSF predominance is expected, as EVs are a primary etiological agent of meningitis [31–35].

Although previous studies of E11 detection in 2022 were linked to hepatitis [9, 10], only 1 case of E11-associated hepatitis was reported in this study. Of interest, all 4 hepatitis-related infections reported here occurred in 2022 and were linked to E7, E11, E21, and CVB3. Additionally, while CVB4 and CVB5 have been associated with myocarditis in earlier reports [11], only a few cases of myocarditis involving these types were reported. Nonetheless these findings demonstrate the diverse clinical spectrum of EV infections.

Interestingly, E18 emerged as the most reported type in 2023, and was frequently associated neurological infections and commonly detected in CSF, accounting for 20% of all infections during the first 8 months of that year. E18 has recently been linked to an outbreak in Hungary associated with neurological outcomes among neonates [12, 13]. Additional studies are needed to assess the factors underlying the emergence of this type in 2023.

Lastly, paralysis was reported among 22 nonpolio EV infections, the majority linked to EV-D68 (n = 4) and EV-A71 (n = 3). As nonpolio EVs are not reported through the World Health Organization´s international health regulation mechanisms, the true incidence of paralysis due to these infections is unknown. A recent study describing data over 21 years shows a detection rate of 6.9% for nonpolio EVs among paralysis cases [36]. These data therefore highlight the clinical and public health burden posed by nonpolio enteroviruses and emphasize the necessity for comprehensive surveillance of nonpolio paralysis cases.

While our study provides valuable insights into the epidemiology of EV infections in Europe, pre- and postpandemic, several limitations should be considered. The aim of this study was to understand the epidemiological and clinical characteristics of EV infections in the years 2018 through 2023 in Europe. For this we relied on data reported by hospitals and public health organizations through ECDC official contact points and ENPEN. Nonpolio enterovirus surveillance is not standardized across Europe, and EV infections are not notifiable in most countries [8]. This lack of standardization resulted in substantial variations in testing practices, case definitions, and reporting standards among institutes and countries.

General increased use of syndromic testing, particularly for respiratory and neurological infections, may have influenced the EV detection rates. As these panels often include EVs as a target, their implementation could have led to a broader EV identification, especially in CSF and respiratory specimens. Additionally, type-specific polymerase chain reaction (PCR) testing for types such as EV-D68 and EV-A71 may have further contributed to the increased detection. Therefore, changes in testing practices and diagnostic algorithms over time likely influenced the observed epidemiology of EVs in this study.

In this study, only 64% of EV-positive samples were subject to typing, which varied per institute/country and was probably influenced by selection factors such as low viral load, clinical presentation, specimen type. As previously shown, most EV testing is conducted in hospital setting [7, 37]. Consequently, only cases presenting to hospital were reported, which likely introduced a bias towards more severe cases. Unfortunately, due to the European General Data Protection Regulation (GDPR), data were sometimes limited or could only be reported in aggregated format, particularly with respect to clinical presentation, with only 10 of 28 participants reporting clinical data. Finally, 42% of all analyzed infections came from France, which reported 4822 infections with clinical and epidemiological data. However, excluding the French data did not result in changes to observed epidemiological patterns, suggesting that our findings remain robust. As sequence data was not collected here, further studies on the evolution of the 10 most commonly reported types could not be conducted.

In conclusion, this study offers valuable insights into the evolving epidemiological and clinical landscape of EV infections in Europe between 2018 and 2023. The results emphasize the widespread circulation of EV types, particularly among young children, and their association with severe outcomes, including neurological infections. The knowledge on type-specific (temporal/clinical) patterns over a longer period could serve as an early warning system for the emergence of specific types, such as E30 or EV-D68, in future years. For this, data need to be collected and shared in a standardized timely manner, as advocated by ENPEN, to assess the disease burden [3].

## Supplementary Material

jiaf179_Supplementary_Data

## Supplementary Data

Supplementary materials are available at *The Journal of Infectious Diseases* online (http://jid.oxfordjournals.org/). Supplementary materials consist of data provided by the author that are published to benefit the reader. The posted materials are not copyedited. The contents of all supplementary data are the sole responsibility of the authors. Questions or messages regarding errors should be addressed to the author.

## Notes

***European Non-Polio Enterovirus Network (ENPEN) study collaborators***: Jan Albert, Nancy Allen, Laurent Andreoletti, Sowsan Atabani, Christelle Auvray, Aldert Bart, Carla Berengua, Natasa Berginc, Maxime Bisseux, Hanneke Boon, Ana Bordes-Benitez, Andrew Bosworth, Sindy Böttcher, Maria Cabrerizo, Annapaola Callegaro, Cristina Calvo, Benjamin Canning, Marianne Coste-Burel, Karen Couderé, Jean-Marie Delarbre, Sabine Diedrich, Susanne Dudman, Robert Dyrdak, Julien Exinger, Eric Farfour, Maria Dolores Fernandez-Garcia, Jacky Flipse, Vincent Foulongne, Kristina Træholt Franck, Floriane Gallais, Magali Garcia, Irina Georgieva, Géraldine Gonfrier, Clémence Guillaume, Almudena Gutierrez-Arroyo, Cécile Henquell, Marijke Hooghiemstra, Elisabeth Huijskens, Anne J. Jääskeläinen, Marion Jeannoël, Marine Jourdain, Elisabeth Toverud Landaas, Kathrin Keeren, Eduardo Lagarejos, Gisèle Lagathu, Sylvie Larrat, Cristian Launes, Mouna Lazrek, Caroline Lefeuvre, Véronique Lemée, Quentin Lepiller, Marianne Leruez, David Leyssene, Anne Sophie L'Honneur, Bruno Lina, Marie Louchet Ducoroy, Maja M. Lunar, Jean-Michel Mansuy, Stéphanie Marque Julliet, Gina Mcallister, C. Patrick McClure, Antonio Medina-Claros, Gregoria Megias, Sofie Elisabeth Midgley, Audrey Mirand, Richard Molenkamp, Milagrosa Montes, Antonio Moreno-Docón, Carmen Munoz-Almagro, Jean-Benjamin Murat, Jean-Luc Murk, Ana Navascues-Ortega, Maria Carmen Nieto-Toboso, Laetitia Ninove, Marije Oosting, Eider Oñate, Jordi Pacaud, Coralie Pallier, Elena Pariani, Laura Pellegrinelli, Mercedes Pérez-Ruiz, Sylvie Pillet, Léa Pilorgé, Luis Piñeiro, Mario Poljak, Birgit Prochazka, Emeline Riverain, Sylvie Rogez, Maud Salmona, Kenda Saloum, Erik Schaftenaar, Cécile Schanen, Manuel Schibler, Isabelle Schuffenecker, Karl Stefic, Petri Susi, Caroline M.A. Swanink, Charlotte Tellini, Anne Lise Toyer, Sara Colonia Uceda Renteria, Juan Valencia-Ramos, Freek B. van Loenen, Inge van Loo, Véronique Venard, Jaco J. Verweij, Karin J. von Eije, Tytti Vuorinen, Elke Wollants, Laura Zanetti.

***Acknowledgments.*** We thank the European Centre for Disease Prevention and Control (ECDC) internal response team members for their contribution to the discussions with the countries and for providing technical support such as epidemic intelligence and outbreak response. We thank the technicians and clinical staff of the clinical microbiology departments and laboratories that have contributed to this study for testing and submitting samples and providing clinical data to the surveillance of enterovirus and the study. Geneva University Hospitals, Geneva, Switzerland acknowledges Francisco-Javier Pérez-Rodríguez who supervised the typing of the Swiss cases.

***Author contributions.*** K. S. M. B., H. H., T. K. F., A. I., L. A., C. K. J., and E. K. B. contributed to study design. K. S. M. B., E. K. B., A. I., and S. d. S. collected and analyzed the data that were discussed with, interpreted by, and finalized with H. H., T. K. F., C. K. J., and E. V. The analysis in RStudio was done by S. d. S., who also wrote the first drafts of the manuscript in collaboration with E. V. The ENPEN study collaborators consisted of representatives working in each participant country at national and regional levels; each country contributed to creating the corresponding dataset, collecting and verifying the requested information from 2018 through 2023. The manuscript was critically commented on by a working group that comprised K. S. M. B., H. H., T. K. F., A. I., L. A., C. K. J., E. V., and E. K. B. and representatives of each country, who checked and approved the final version.

***Disclaimer*.** The views and opinions expressed herein do not state or reflect those of ECDC. ECDC is not responsible for the data and information collation and analysis and cannot be held liable for conclusions or opinions drawn.

***Financial support.*** This work was supported by the national surveillance enterovirus programs of the partcipating insititutes with specific funding from the Centre National de Référence des Enterovirus-Parechovirus funded by the French National Public Health Network, Santé Publique France (to CHU Clermont-Ferrand, Centre national de reference des enterovirus et parechovirus – laboratoire coordonnateur, Clermont-Ferrand, France); the Instituto de Salud Carlos III (grant numbers PI18CIII/00017 and PI22CIII/00035 to Enterovirus and Viral Gastroenteritis Lab, National Centre for Microbiology, Instituto de Salud Carlos III and the Spanish Research Networks Consortium of Epidemiology and Public Health, Madrid, Spain); the UK Clinical Virology Network (to Nottingham University Hospitals National Health Service (NHS) Trust, Nottingham, United Kingdom and Birmingham University Hospitals Trust, Birgmingham, United Kingdom for typing); and the Helsinki University Hospital (HUS) Diagnostic Center, Helsinki, Finland (grant number TYH2024104 to University of Helsinki, Helsinki, Finland); Emiel Vanhulle is supported by the European Centre for Disease Prevention and Control Fellowship Programme.
