# Supplementary material for: Epidemiological and Clinical Insights into Enterovirus Circulation in Europe, 2018–2023: A Multicenter Retrospective Surveillance Study
Source: J Infect Dis. 2025 Apr 4;232(1):e104–15. doi: 10.1093/infdis/jiaf179 (PMC12308651; doi:10.1093/infdis/jiaf179)
Supplement: jiaf179_Supplementary_Data [file jiaf179_supplementary_data.zip › Manuscript_SdS_Tables_supp_clean_final.docx]

## **Supplementary table S1. Reported data on specimens tested, EV-positive, subjected to typing and successfully typed per institute (n=28) from 16 countries, 2018-2023.**

| **Country** | **Reporting institution** | **Tested** | **EV Positive** | | **Subjected to typing** | | **EV typed** | | |
| --- | --- | --- | --- | --- | --- | --- | --- | --- | --- |
|  |  | **n** | **n** | **% of tested** | **n** | **% of positives** | **n** | **% of positives** | **% of subjected typing** |
| Austria | AT01 | 2341 | 366 | 15.6% | 316 | 86.3% | 290 | 79.2% | 91.8% |
| Belgium | BE02 | 18821 | 3160 | 16.8% | 2245 | 71.0% | 1948 | 61.6% | 86.8% |
| Bulgaria | BG01 | 1246 | 168 | 13.5% | 31 | 18.5% | 27 | 16.1% | 87.1% |
| Denmark | DK01 | NA | 3620 | - | 2402 | 66.4% | 1434 | 39.6% | 59.7% |
| England, UK | UK04 | 17182 | 900 | 5.2% | 314 | 34.9% | 199 | 22.1% | 63.4% |
|  | UK09 | 6949 | 1049 | 15.1% | 51 | 4.9% | 40 | 3.8% | 78.4% |
| Finland | FI01 | 19546 | 428 | 2.2% | 97 | 22.7% | 97 | 22.7% | 100.0% |
|  | FI03 | 3221 | 33 | 1.0% | 9 | 27.3% | 9 | 27.3% | 100.0% |
| France | FR01* | 276313 | 11531 | 4.2% | 8819 | 76.5% | 7800 | 67.6% | 88.4% |
| Germany | DE01 | 8925 | 1305 | 14.6% | 1189 | 91.1% | 1048 | 80.3% | 88.1% |
| Italy | IT03 | 17229 | 494 | 2.9% | 301 | 60.9% | 114 | 23.1% | 40.0% |
| Netherlands | NL03 | 6928 | 736 | 10.6% | 412 | 56.0% | 299 | 40.6% | 72.6% |
|  | NL04 | NA | NA | - | NA | - | 253 | - | - |
|  | NL05 | 29607 | 373 | 1.3% | 359 | 96.2% | 220 | 59.0% | 61.3% |
|  | NL18 | 7509 | 805 | 10.7% | 71 | 8.8% | 59 | 7.3% | 83.1% |
|  | NL19** | 1760 | 275 | 15.6% | NA | - | NA | - | - |
|  | NL20 | 3262 | 269 | 8.2% | 204 | 75.8% | 166 | 61.7% | 81.4% |
|  | NL21 | 1070 | 241 | 22.5% | 123 | 51.0% | 107 | 44.4% | 87.0% |
|  | NL22 | 2263 | 85 | 3.8% | 29 | 34.1% | 27 | 31.8% | 93.1% |
| Norway | NO02a | 3802 | 723 | 19.0% | 404 | 55.9% | 353 | 48.8% | 87.4% |
|  | NO02b | 4450 | 89 | 2.0% | 23 | 25.8% | 19 | 21.3% | 82.6% |
| Scotland, UK | UK05 | NA | NA | - | 559 | - | 340 | - | 60.8% |
| Slovenia | SI01 | 10640 | 498 | 4.7% | 498 | 100.0% | 265 | 53.2% | 53.2% |
|  | SI02 | 2555 | 112 | 4.4% | 81 | 72.3% | 52 | 46.4% | 64.2% |
| Spain | ES02 | 41553 | 864 | 2.1% | 561 | 64.9% | 261 | 30.2% | 46.5% |
|  | ES05* | NA | 2545 | - | 2168 | 85.2% | 1652 | 64.9% | 76.2% |
| Sweden | SE01 | 65105 | 2305 | 3.5% | NA | - | NA | - | - |
| Switzerland | CH01** | 11377 | 291 | 2.6% | 2 | 0.7% | 2 | 0.7% | 100.0% |
| Total |  | 563654 | 33265 | 5.9% | 21268 | 63.9% | 17081 | 51.3% | 80.3% |

* Institutes FR01, and ES05 reported data for 41 and 9 institutes respectively

**Only background testing data was reported from two institutes (NL19 and CH01). Detailed clinical and epidemiological information was not reported; indicated as NA: not applicable.

## **Supplementary table S2.** **Reported epidemiological and clinical data on enteroviruses typed per institute (n=28) from 16 countries, 2018-2023. Data reported for > 25% of cases are indicated as Yes. Data reported for <25% of cases are indicated as No/incomplete.**

| **Country** | **Reporting institution** | **Month reported** | **Specimen type reported** | **Most common specimen type (%)** | **Age reported** | **Clinical sign reported** | **Typing method** |
| --- | --- | --- | --- | --- | --- | --- | --- |
| Austria | AT01 | No/incomplete | Yes | Fecal (60%) | Yes | No/incomplete | VP1-seq |
| Belgium | BE02 | Yes | Yes | CSF (55%) | Yes | No/incomplete | VP1-seq |
| Bulgaria | BG01 | Yes | No/incomplete | Unreported | No/incomplete | No/incomplete | Virus neutralization/ VP1-seq*** |
| Denmark | DK01 | Yes | Yes | Fecal (37%) | Yes | No/incomplete | VP1-seq**** |
| England, UK | UK04 | Yes | Yes | Vesicle (40%) | Yes | Yes | VP1-seq |
|  | UK09 | Yes | Yes | CSF (58%) | Yes | Yes | VP1-seq |
| Finland | FI01 | No/incomplete | No/incomplete | Unreported | Yes | No/incomplete | VP1-seq |
|  | FI03 | Yes | Yes | Respiratory (82%) | Yes | Yes | VP1-seq |
| France | FR01* | Yes | No/incomplete | Unreported | Yes | No/incomplete | VP1-seq |
| Germany | DE01 | Yes | Yes | Fecal (93%) | Yes | Yes | VP1-seq |
| Italy | IT03 | No/incomplete | Yes | Respiratory (96%) | Yes | Yes | VP1-seq |
| Netherlands | NL03 | Yes | Yes | Fecal (76%) | No/incomplete | No/incomplete | VP1-seq |
|  | NL04 | No/incomplete | Yes | Fecal (35%) | Yes | Yes | VP1-seq |
|  | NL05 | Yes | Yes | Respiratory (43%) | Yes | No/incomplete | VP1-seq |
|  | NL18 | Yes | Yes | Fecal (81%) | Yes | Yes | VP1-seq |
|  | NL19** | NA | NA | NA | NA | NA | VP1-seq |
|  | NL20 | Yes | Yes | Blood (43%) | Yes | Yes | VP1-seq |
|  | NL21 | Yes | Yes | Fecal (72%) | Yes | No/incomplete | VP1-seq |
|  | NL22 | Yes | Yes | Fecal (81%) | Yes | No/incomplete | VP1-seq |
| Norway | NO02a | Yes | Yes | Fecal (58%) | Yes | No/incomplete | VP1-seq |
|  | NO02b | Yes | Yes | Blood (50%) | Yes | Yes | VP1-seq |
| Scotland, UK | UK05 | Yes | No/incomplete | Unreported | No/incomplete | No/incomplete | VP1-seq |
| Slovenia | SI01 | Yes | Yes | Respiratory (96%) | Yes | Yes | VP1-seq |
|  | SI02 | Yes | Yes | CSF (100%) | Yes | No/incomplete | VP1-seq |
| Spain | ES02 | Yes | Yes | Respiratory (83%) | No/incomplete | No/incomplete | VP1-seq |
|  | ES05* | Yes | Yes | Respiratory (42%) | Yes | Yes | VP1-seq |
| Sweden | SE01 | Yes | Yes | Respiratory (74%) | Yes | No/incomplete | VP1-seq |
| Switzerland | CH01** | NA | NA | NA | NA | NA | VP1-seq |

* Institutes FR01, and ES05 reported data for 41 and 9 institutes respectively

**Only background testing data was reported from two institutes (NL19 and CH01). Detailed clinical and epidemiological information was not reported; indicated as NA: not applicable.

***The Bulgarian laboratory uses primarily virus neutralization, but a selection of samples is subjected to VP1 sequencing as well

****The Danish laboratory used VP2/4-sequencing from 2018-2022, however it implemented VP1-sequencing in 2023.
