# Supplementary material for: Epidemiological and Clinical Insights into Enterovirus Circulation in Europe, 2018–2023: A Multicenter Retrospective Surveillance Study
Source: J Infect Dis. 2025 Apr 4;232(1):e104–15. doi: 10.1093/infdis/jiaf179 (PMC12308651; doi:10.1093/infdis/jiaf179)
Supplement: jiaf179_Supplementary_Data [file jiaf179_supplementary_data.zip › Suppl figure legends.docx]

## **Supplementary Figure legends**

**Supplementary Figure 1.** Diagram of the information collected: background testing and typing data with the number of cases with reported information on month of detection, the patient-age, specimen type tested, and the clinical presentation of the cases, from 2018 to 2023. Map shows the geographical distribution of countries (indicated in blue, n=16) with reporting institutions in Europe that provided data on EV testing and typing between 2018 and 2023.

**Supplementary Figure 2**. Enterovirus detection patterns in terms of specimen type (A), age group (B) and clinical presentations (C) associated with the ten most commonly reported EV types per year, 2018-2023. The color intensity in the heat maps is based on the natural logarithm of the fraction of the case numbers associated with the specified variable over all case numbers by that type adding up to 100%. Black regions represent either zero infections or no data.
