# Supplementary figures and images for: Epidemiological and Clinical Insights into Enterovirus Circulation in Europe, 2018–2023: A Multicenter Retrospective Surveillance Study
Source: J Infect Dis. 2025 Apr 4;232(1):e104–15. doi: 10.1093/infdis/jiaf179 (PMC12308651; doi:10.1093/infdis/jiaf179)

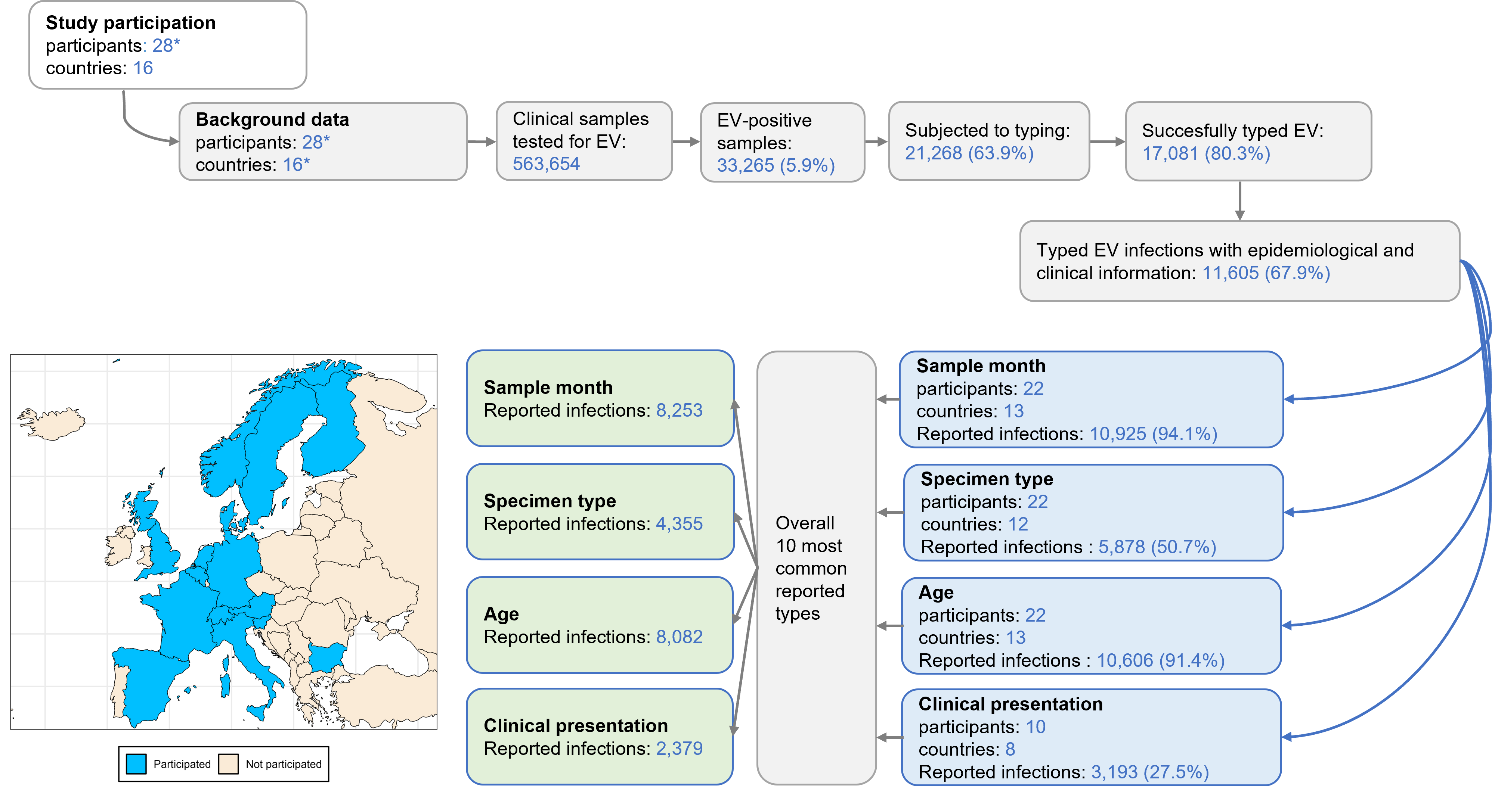

Supplement: jiaf179_Supplementary_Data [file jiaf179_supplementary_data.zip › Manuscript_SdS_Figure_suppl 1_png.png]
